# Brazilian Jiu Jitsu players’ motivations to train

**DOI:** 10.3389/fpsyg.2023.1240351

**Published:** 2023-09-19

**Authors:** Terrance L. Tarver, Jacob J. Levy

**Affiliations:** Department of Psychology, University of Tennessee, Knoxville, TN, United States

**Keywords:** sport participation, self-determination theory, martial arts, combat sports, exercise motivation

## Abstract

Combat sports, such as Brazilian Jiu Jitsu (BJJ), require intense physical, mental, and emotional tasking within its training. With the degree of difficulty ingrained within the sport, many participants that once were intrigued by the sport may lose this interest and enjoyment if their goals are not met. The purpose of this study was to examine the relative strength of sport motivations among BJJ players. Participants included 228 BJJ athletes varying in levels of sport participation experience. Grounded in Self-Determination Theory, participants were assessed on five motives for sport participation including: fitness, appearance, competence, social, and interest/enjoyment. Motives related to interest/enjoyment, competence, and fitness, were rated relatively higher; and appearance and social were rated relatively lower regarding participants’ motivation for BJJ participation. Analyses were also conducted related to athletes’ years of experience and competitive level of participation (i.e., hobbyist or non-competitor to those who compete on a regular basis) There was a significant effect of competence and interest/enjoyment motivators among competitive BJJ players, regardless of years of experience in the sport. Findings from this study could aid coaches, sport clinicians, and sport psychologists in working with BJJ players by focusing their training on the motivators that are most appealing to these athletes.

## Introduction

Brazilian Jiu Jitsu (BJJ) is a grappling combat sport that mainly utilizes chokeholds and joint locks. Over the past 30 years, interest in BJJ participation has grown tremendously. While BJJ is a prominent sport in Brazil, it has garnered attention and interest worldwide (Andreato et al., 2017). This rise has been found in a multitude of countries such as the United Kingdom where the number of BJJ gyms has grown exponentially from 12 in 2009 to a staggering 320 in 2020 (Sugden, 2021).

Motivation is the force that drives people to initiate and sustain their effort toward their goals (Liu et al., 2015). It is instrumentally critical to sport participation in athletes because it is a pivotal element in determining the actions and efforts that they take within their sport (Ryan and Deci, 2000; Rintaugu et al., 2014; Vink and Raudsepp, 2018). Sport practitioners have found motivation to be one of the most difficult and misconstrued concepts to define within psychology (Williams and Krane, 2015). This is a result of many biological and environmental determinants that influence motivation for each athlete. Motivation is defined as “a combination of an individual’s desire to take action, also known as their drive, and the direction in which these said actions are aimed” (Weinberg and Gould, 2007, p. 52). Motivation can partly be determined by the variation of an athlete’s behavioral patterns as well as the quantity and quality of their motivation (Duda, 2001, 2005). For example, it has been found that athletes who are more intrinsically motivated are more likely to continue their participation in their sport (Sarrazin et al., 2002), especially if they are participating in an athlete-centered (as opposed to outcome focused) climate—one that is perceived as caring, autonomy-supportive, empowering, and task-involving (Fry and Moore, 2019).

Motivation can be impacted by several internal and external factors that an athlete may experience. The relation between the athlete and these factors is of vital importance for the well-being of athletes because of the impact it may have on the athlete’s motivation, which in turn influences an athlete’s ability to achieve the desired level of performance for their sport (Ryan and Deci, 2000). Research findings indicate athletes experience more satisfaction and joy from their sport participation when it is perceived as intrinsically motivating, or for the enjoyment of participation (Deci and Ryan, 2000). However, separable rewards such as athletic scholarships and professional sports careers have the potential to hinder this intrinsic motivation (Deci, 1971). While these derivatives of motivation have been analyzed through many theories, multiple researchers and sport practitioners have utilized self-determination theory to access the motivation of athletes.

Self-determination theory (SDT) bases its origins off the seminal work of Deci and Ryan (1985). It is a macro theory of human motivation that differentiates between different types of motivation depending on the motives, goals, and actions that influence the motivation. SDT further categorizes these different reasons for an athlete’s actions on a continuum of behavioral regulations with some having elements of both intrinsic and extrinsic motivation (Ryan and Deci, 2000; Amorose et al., 2016). Intrinsic actions are considered those in which an athlete engages in an activity for the pleasure and satisfaction derived from the activity itself. Extrinsically motivated athletes engage in an activity to attain some separable outcome such as for a medal, fame, or financial reward. The extent to which extrinsic motivation is self-determined allows this motivation to be explained along a continuum (Vansteenkiste et al., 2010; Taylor, 2015). This continuum places these motivations from the most extrinsic (external regulation) to the most intrinsic (intrinsic regulation).

The sport of BJJ relies heavily on positioning, leverage, and technique, and relies less on physical attributes such as size, strength, speed, and quickness (Gracie and Danaher, 2003). With the focus of the sport resting heavily on the acquisition of submission holds and positions, BJJ requires a keen level of competence to not only defeat an opponent, but to also elude an opponent that is aiming to place a player in an uncompromising position (Ovretveit et al., 2018). A mishap in applying a technique could create an unwelcoming experience for a non-competent BJJ player. While BJJ provides an opportunity for athletes to gain competence in a new sport, it also allows athletes to learn techniques to utilize in their everyday lives as well. BJJ has long been regarded as a great self-defense art that provides competence in using leverage to defend oneself (Jeon, 2020).

Although many BJJ athletes begin their training out of sheer interest in the sport, some continue to participate in part to the social connections that they make within their gym. BJJ athletes have noted that the social environment and connections made during their training have had a significant impact on their comfortability within their gym as well as providing many mental health benefits such as positive coping, resilience, and perseverance (Reusing, 2014; Chinkov and Holt, 2016; Mickelsson, 2021; Sugden, 2021). Research has also found that social connections to others deemed as vital to a BJJ athlete’s gym is correlated with a higher probability of continued participation as well as stronger feelings of connectedness to others (Rodrigues et al., 2019). These social connections and environment that BJJ players participate in foster a therapeutic, comforting, and encouraging environment to thrive (Willing et al., 2019).

BJJ athletes may also find encouragement to engage in their sport from extrinsic motivators as well, such as the physical appearance and fitness benefits that BJJ can provide. BJJ has been found to improve cardiovascular functioning, strength, bone mineral density, flexibility, and nutrition, as well as reduce blood pressure and body fat (Burke et al., 2007; Tsang et al., 2008; Boguszewski et al., 2014; Kim et al., 2014). These benefits may also aid in enhancing BJJ athletes’ physical appearance as well. Research has even shown that it is possible to experience these benefits with as little as 2 h of BJJ training per week (Lorenco-Lima et al., 2020).

Motivation for BJJ players may also come from the achievement of belt progression. Within BJJ, players progress through a belt ranking consisting of the colors white, blue, purple, brown, and black, to designate their skill level within the sport. While this progression can provide motivation, it may also lead to higher dropout rates among players. For example, a blue belt within a gym, while still considered a relative novice within the sport, may have additional expectations bestowed upon them with their first belt promotion. Blue belts may feel that they must be better than all white belt practitioners while also being competitive against more advanced belts (Ovretveit et al., 2018). These expectations can lead to negative outcomes and behaviors such as performance-avoidance tendencies. With these heightened expectations, many blue belts may feel that they can no longer perform at a desired level, which may lead to drop out. With the multitude of motivators that can draw athletes to BJJ, there is a dearth of research that investigates what motivators BJJ athletes value most.

### Current study

The purpose of the current study was to examine the relative strength of sport motivations among Brazilian Jiu Jitsu players consisting of both intrinsic and extrinsic motivators. We posited and tested the following hypotheses:

*H1:* BJJ athletes would rate the intrinsic motivators of interest/enjoyment, competence, and social significantly higher than the extrinsic motivators of fitness and appearance. Rationale: The physical and high cardio nature of BJJ creates a difficult environment for training and participation. Therefore, BJJ athletes could be more likely to find motivators that enhance motivation outside of receiving awards and praise.

*H2:* The number of years BJJ training and competitive level of BJJ participation (i.e., hobbyist, competitive hobbyist, regular amateur competitor, elite amateur competitor, and professional athlete) would be positively related to intrinsic motivators of interest/enjoyment, competence, and social but not for the extrinsic motivators of fitness and appearance. Rationale: The researchers posit those participants that have engaged in BJJ participation longer and who competed more regularly would espouse more intrinsically oriented motivators than those who have participated for a significantly less amount of time or competed less.

*H3:* Participants who regularly engaged in BJJ competition (i.e., amateur, elite amateur and professional athletes) would rate the intrinsic motivators of interest/enjoyment, competence, and social higher than those BJJ players that participated primarily for enjoyment and fitness (i.e., hobbyists and competitive hobbyists).

## Materials and Methods

### Participants

This study included 228 BJJ players. Most participants resided within the United States with 165 (72.4%) followed by Canada with 12 (5.3%); the United Kingdom with 10 (4.4%); Ireland with seven (3.1%); Australia with six (2.6%); Germany had four (1.8%); and Sweden with three (1.3%). Three countries (Northern Iran, the United Arab Emirates, and Thailand) had two (0.9%) while 15 countries (Poland, Pakistan, Norway, Switzerland, Ukraine, Italy, South Africa, Luxembourg, Belgium, Singapore, New Zealand, Romania, France, Austria, and Netherlands) had one (0.4%). Regarding race, the majority of participants (*n* = 183; 80.3%) identified as White, followed by Asian or Asian American (*n* = 18, 7.9%), Hispanic (*n* = 13, 5.7%), Interracial (*n* = 12, 5.3%), and African American or Black (*n* = 2, 0.9%). Regarding sex, the majority of participants identified as male (*n* = 186, 81.6%); with 40 (17.5%) identifying as women, and two (0.9%) identifying as other. Participants were asked to disclose their ages within set ranges. The majority of participants fell within the 25–34 age range with 86 (37.7%) followed by the 35–44 age range with 68 (29.8%), the 45–54 age range with 40 (17.5%), the 18–24 age range with 28 (12.3%) and the 55–64 age range with six (2.6%). All recruitment messages and studies measures were presented solely using the English language; thus the possible sample was limited to those who could read English.

### Procedures

The study was reviewed and approved for human subjects by the authors’ Institution Review Board (UTK IRB-20-05816-XM). This current study is based on a sub-set of data collected as part of larger investigation of combat sport athletes exercise behavior, mental health, and exercise motivation. Participants were recruited through social media groups such as Facebook and other combat sport web groups. Permission from administrators of these social media groups and websites was granted prior to research solicitation. The posts included a link to the secured survey within the Survey Monkey. The survey began with an informed consent statement, and participants indicated their consent to participate by checking the “I agree” button (this is the only required response needed to submit the survey). Next, participants were asked to respond to the questionnaires. Participation was voluntary and anonymous.

### Instrumentation

The participants first filled out a demographic form as a part of the survey. The demographic asked for non-identifying demographic information such as the participants’ current age, sex, race, country of origin, and region (if country of origin was the US). This information from this demographic form was used as descriptive data for this study.

#### Level of participation

Participants were asked to indicate their level of participation within BJJ within the survey. This information was garnered by having participants mark one of the following levels of participation: hobbyists (i.e., train exclusively for recreation and fitness), competitive hobbyists (i.e., train mostly for recreation and fitness, but occasionally compete in BJJ), amateur athletes (i.e., train for the purposes of regularly competing as an amateur BJJ player), elite amateur athletes (i.e., train to compete at highest level of amateur competition—for example, Olympic, national, and/or international competition); and professional combat sport athletes. Regarding competitive level of participation, 60 (26.3%) were hobbyists; 97 (42.5%) participants identified as competitive hobbyists, 49 (21.5%) as amateur athletes, 17 (7.5%) as elite amateur athletes, and 5 professional combat sport athletes (2.2%). For the purposes of assessing meaningful comparisons among level groups, we reallocated participants into one of three groups: hobbyists (*n* = 60, 26.3%); competitive hobbyists (*n* = 97, 42.5%), and competitors (i.e., those train to regularly compete in BJJ at the amateur, elite amateur, and professional levels—*n* = 71, 31.1%).

#### Motivation

The Motives for Physical Activity Measure-Revised (MPAM-R; Ryan et al., 1997) was used to assess the strength of motives for athletes’ participation in BJJ. The five motives measured are: (1) fitness—being physically active out of the desire to be physically healthy and to be strong and energetic; (2) appearance—being physically active in order to become more physically attractive, to have defined muscles, to look better, and to achieve or maintain a desired weight; (3) competence—being physically active because of the desire just to improve at an activity, to meet a challenge, and to acquire new skills; (4) social—being physically active in order to be with friends and meet new people; and (5) interest/enjoyment—being physically active just because it is fun, makes you happy, and is interesting, stimulating, and enjoyable. The scale has been used to predict various behavioral outcomes, such as attendance, persistence, or maintained participation in some sport or exercise activity, or to predict mental health and well-being. This scale was created on the foundations of Self-Determination Theory and the basic psychological needs of competence, relatedness, and autonomy. The scales motives have been found to be associated with intrinsic and extrinsic motivation with the interest/enjoyment, social, and competence scales relating to intrinsic motivation while the appearance and fitness scales relating to extrinsic motivation. Internal consistency estimates for the current sample across the five scales were consistent with those reported in the normative sample (Ryan et al., 1997) and indicative of good to very good reliability. Coefficient alphas for the current sample were as follows: fitness—alpha = 0.86, appearance—alpha = 0.91, competence—alpha = 0.87, social—alpha = 0.79, and interest/enjoyment—alpha = 0.83.

### Data analysis

Statistical analyses were performed using Version 27 of the Statistical Package for Social Science (SPSS). A one-sample *t*-test was conducted to test the mean rating of five motive subscales. Multiple regression analyses were used to test if the number of years trained significantly predicted participants’ ratings of the motivators. Finally, a one-way between subjects MANOVA was conducted to compare athletes’ level of participation on their motivation ratings.

## Results

The study was presented as a two-page survey *via* Survey Monkey (an online data collecting platform). The first page of the study consisted of demographic questions as well as level of participation of clients. Two hundred twenty-seven participants (99.6%) responded to all questions regarding country of origin, gender, age, and race, as well as all of the items on the MPAM-R.

To test *H1*, a one-sample *t*-test was conducted to test the mean rating of five motive subscales (appearance, competence, fitness, interest/enjoyment, and social) relative to sample mean of all items (overall *M* = 5.54, range 1–7) on the MPAM-R. Three subscales were found have significantly higher endorsements relative to overall sample mean: interest/enjoyment, *M* = 6.40; SD = 0.73, *t*(227) = 17.78, *p* < 0.001, Cohen’s *d* = 0.73; competence, *M* = 6.27; SD = 0.89, *t*(227) = 12.21; *p* < 0.001, Cohen’s *d* = 0.90, and fitness, *M* = 5.95; SD = 1.13, *t*(227) = 5.47, *p* < 0.001, Cohen’s *d* = 1.13. Two subscales had significantly lower endorsement that overall sample mean: appearance, *M* = 4.59; SD = 1.46, *t*(227) = −9.76; *p* < 0.001, Cohen’s *d* = 1.46; and social, *M* = 4.47; SD = 1.28, *t*(227) = −12.62; *p* < 0.001, Cohen’s *d* = 1.28. Table 1 presents the mean rating and relative difference from the overall sample mean for all items.

**Table 1:** One sample *t* test comparing relative strength of MPAM-R item responses (in descending order of mean score).

Item	*M*	SD	*t*(227)	*p*
Like to do this activity	6.64	0.78	21.34	<0.001
Enjoy this activity	6.61	0.89	18.04	<0.001
Find the activity stimulating	6.56	0.85	18.05	<0.001
Think it is interesting	6.54	0.85	17.91	<0.001
Makes me happy	6.54	0.98	15.45	<0.001
Want to get better at this activity	6.51	0.88	16.64	<0.001
It’s fun	6.47	1.00	13.98	<0.001
Like the challenge	6.41	1.03	12.78	<0.001
Want to obtain new skills	6.29	1.13	10.00	<0.001
Like activities that physically challenge me	6.26	1.16	09.43	<0.001
Want to improve existing skills	6.26	1.25	08.69	<0.001
Maintain physical health and well-being	6.19	1.25	07.83	<0.001
Want to be physically fit	6.14	1.18	07.76	<0.001
Maintain physical strength for a healthy life	6.12	1.31	06.69	<0.001
Keep my current skill level	6.07	1.45	05.54	<0.001
Like activities that are physically challenging	6.04	1.38	05.56	<0.001
Want to improve cardiovascular fitness	5.72	1.49	01.83	0.07
Enjoy spending time with others doing the activity	5.64	1.60	00.95	0.34
Like to be with others who are interested in the activity	5.60	1.62	00.53	0.60
Want to have more energy	5.56	1.72	00.19	0.85
Like the excitement of participation	5.44	1.65	−00.93	0.36
Want to lose weight so I look better	5.03	1.92	−04.00	<0.001
Want to improve my body shape	4.88	1.78	−05.63	<0.001
Want to be with my friends	4.87	1.90	−05.30	<0.001
Want to improve my appearance	4.64	1.93	−07.06	<0.001
Define my muscles so I look better	4.49	1.90	−08.32	<0.001
Want to meet new people	4.35	1.93	−09.34	<0.001
Want to be attractive to others	4.07	2.00	−11.13	<0.001
Physically unattractive if I do not	2.99	2.15	−17.90	<0.001
Friends want me to do it	1.92	1.53	−35.76	<0.001

Item range 1–7 indicating reasons participants participate in BJJ with 1 being not at all true for them, to 7 being very true for them. Mean of all items for sample was 5.54 which served as the test value for the one-sample *t*-test.

To address *H2*, five multiple linear regressions were calculated to predict if the number of years trained and level of training (operationalized as 1 = hobbyist, 2 = competitive hobbyist, and 3 = regular competitor) predicted participants’ ratings of the motivators of interest/enjoyment, competence, appearance, social, and fitness. Follow-up simple regression were then used to examine the effect years of experience and training level separately.

Using the enter method it was found that years of experience and training level explained a significant amount of the variance in the value of competence, *F*(2, 225) = 3.91, *p* = 0.022, *R*^2^ = 0.34, *R*^2^_Adjusted_ = 0.25. The simple regression analysis showed that years of experience did not significantly predict value of competence motivators [Beta = 0.06, *t*(227) = 0.90, *p* = 0.37], however training level did significantly predict value of competence motivators [Beta = 0.16, *t*(227) = 2.48, *p* = 0.014].

Regarding interest/enjoyment motivators, the linear combination of years and experience and training level did not explain a significant amount of variance *F*(2.225) = 2.64, *p* = 0.074, *R*^2^ = 0.02, *R*^2^_Adjusted_ = 0.01. The simple regression analysis showed that years of experience did not significantly predict value of interest/enjoyment motivators [Beta = 0.059, *t*(227) = 0.00, *p* = 0.996], however training level did significantly predict value of interest/enjoyment motivators [Beta = 0.15, *t*(227) = 2.27, *p* = 0.024].

Regarding appearance motivators, the linear combination of years and experience and training level did not explain a significant amount of variance *F*(2,225) = 0.27, *p* = 0.768, *R*^2^ = 0.002, *R*^2^_Adjusted_ = −0.007. The simple regression analysis showed that neither years of experience [Beta = 0.01, *t*(227) = 0.12, *p* = 0.903] nor training level [Beta = 0.15, *t*(227) = 0.69, *p* = 0.491] significantly predicted value of appearance motivators.

Regarding social motivators, the linear combination of years and experience and training level did not explain a significant amount of variance F(2,225) = 1.37, *p* = 0.256, *R*^2^ = 0.012, *R*^2^_Adjusted_ = 0.003. The simple regression analysis showed that neither years of experience [Beta = −0.01, *t*(227) = −0.16, *p* = 0.872] nor training level [Beta = 0.11, *t*(227) = 1.65, *p* = 0.109] significantly predicted value of social motivators.

Finally, regarding fitness motivators, the linear combination of years and experience and training level did not explain a significant amount of variance *F*(2,225) = 0.08, *p* = 0.916, *R*^2^ = 0.001, *R*^2^_Adjusted_ = −0.008. The simple regression analysis showed that neither years of experience [Beta = −0.02, *t*(227) = −0.35, *p* = 0.728] nor training level [Beta = −0.01, *t*(227) = −0.18, *p* = 0.859] significantly predicted value of social motivators.

To address *H3*, a multivariate analysis of variance (MANOVA) was used to compare the effect of participant levels of participation (hobbyist, competitive hobbyist, or regular competitor) on their motivation ratings of interest/enjoyment, competence, appearance, fitness, and social. The multivariate test of the differences among the three groups was significant, Pillai’s trace = 0.035, *F*(10,444) = 3.11, *p* < 0.001, partial *η*^2^ = 0.065 (medium effect). Of the univariate tests, competence, *F*(2,225) = 8.57, *p* < 0.001, partial *η*^2^ = 0.071 (medium effect), and fitness, F(2,225) = 5.24, *p* = 0.006, partial *η*^2^ = 0.045 (small effect), were significant at *p* ≤ 0.016 (for three levels). Using the Dunnett’s C method (which does not assume equal variance due to sample size differences among the competitive training levels), each ANOVA for the three groups was tested at the 0.017 level. The only significant (*p* < 0.017) mean difference was between hobbyists (*M* = 5.88, SD = 1.01) and competitive hobbyists (*M* = 6.47, SD = 0.76) on the competence motivators. Regular competitors (*M* = 6.32, SD = 0.88) were different than hobbyists on the competence motivators at the *p* < 0.05 level (shy of the needed significant probability for comparisons among three groups). The univariate tests for interest/enjoyment, F(2,225) = 3.09, *p* = 0.05, partial *η*^2^ = 0.03; appearance, F(2,225) = 0.89, *p* = 0.51, partial *η*^2^ = 0.01; and social, F(2,225) = 2.51, *p* = 0.08, partial *η*^2^ = 0.02 were not significant.

## Discussion

The findings from this study suggest BJJ athletes find both intrinsic and extrinsic motivation to participate in their sport. However, these athletes tend to value the motivators of interest/enjoyment, competence, and fitness relatively stronger than appearance and social motives—especially if they compete in BJJ. Research supports the findings of interest/enjoyment with the recent boom of interest and popularity in BJJ by practitioners and spectators over the past few decades (Blue, 2013; Andreato et al., 2017). The findings also align with the fitness benefits that have been studied within BJJ practice such as reducing body fat and blood pressure as well as improvements in flexibility and cardiovascular functioning (Burke et al., 2007; Tsang et al., 2008; Boguszewski et al., 2014; Kim et al., 2014), while also highlighting the benefits regarding competition. Lean mass within the upper quartile of an athlete’s weight class has been positively correlated with the ability to perform technical actions within a performance (Franchini et al., 2007; Kim et al., 2011; Marinho et al., 2012). These fitness benefits in novel practice and competition bring light to what lead fitness motivation to be in high regards with BJJ players. While interest/enjoyment and fitness were rated relatively high on the MPAM-R, competence was the motivator that participants referred to on multiple occasions.

Competence as motivation for BJJ athletes can be viewed in many aspects of the sport including the objective of the sport. For a competitor to be victorious in a match, the competitor must either submit their opponent or be ahead in points, based on positional dominance, at the end of the bout. Both avenues to victory create a need for BJJ players to be technically sound and possess a vast knowledge of submissions, passes, takedowns, and positions. BJJ players that fail to obtain and maintain competence in their technical ability will find it difficult to progress within the sport. This coincides with the findings of this study in that competition hobbyists and elite amateurs rated competence higher than hobbyists. Both elite amateurs and competition hobbyists compete in BJJ in some capacity within their participation. These athletes must be competent in their skills and techniques to compete with others within their competitive field, which is usually based on belt rank within BJJ, which is also a measure of competence.

Regarding *H2*, we found positive correlations between competitive training level and competence, and interest/enjoyment motivation ratings. Similarly, and related to *H3*, competitive BJJ athletes endorsed higher competence (medium effect) and fitness (small effect) motivators relative those athletes who identify as hobbyists in the sport. However, our hypothesis regarding relations between years of experience and sport motivation were not supported. As BJJ athletes gain competence in their sport, they are recognized by being awarded a higher belt rank within their training (either blue, purple, brown, or black) as a symbol of skill (Ovretveit et al., 2018). To advance to a new belt, BJJ players must exemplify mastery and competence over various techniques and skills to ensure that they are qualified for belt promotion. When BJJ players compete, they do so against other at the same skill or belt level. Thus, BJJ athletes’ engagement in competition, regardless of number of years of sport participation, was found to be more predictive of their motivations for sport participation.

The results of this study also coincide with the basic psychological needs of competence and relatedness. Participants found competence to be a relatively high motivator which can also influence other perceived aspects of their sport. For example, high perceived competence within athletes increases expectations for success, intrinsic motivation, persistence to face and overcome adversity, exerted efforts, such as engagement, effort to master skills, persistence in the face of difficulty, and choice of challenging tasks (Roberts et al., 2007). Relatedness was expressed through the significantly high ratings that participants had for the social motivator. Participants seemed to value connectedness to others within their training environment. This relatedness has been shown to predict indices of self-determination and intrinsic motivation for both sport participation and exercise behavior (Stults-Kolehmainen et al., 2013).

### Practical implications

The findings of this study could aid BJJ instructors in their training techniques for their students. BJJ instructors may take the notion of competence as a motivator and revise their training to emphasize the acquisition and mastery of skills and techniques, instead of emphasizing the progression of belt rank—especially for athletes with goals of competing in BJJ. This type of training can keep BJJ players engaged with their training for a more intrinsic motivator, which could maintain and/or increase participation, even if belt progression is not occurring at the frequency that the BJJ player would hope for.

Sport clinicians and sport psychologists can benefit from the findings of this within their own work with BJJ players. Sport psychologists may find that tailoring their interventions to focus more on the competence that the BJJ player has within their sport could foster more dialogue and a deeper therapeutic relationship between the sport psychologist and the BJJ player. For example, an intervention centered around the BJJ player’s purpose for participation regarding competence could open avenues to further discuss concerns that the BJJ player may have in this area such as self-doubt, performance anxiety, and issues with focusing.

### Limitations

There are a few limitations to this study, starting with the diversity in the participant pool. The majority of participants were White men from the United States. It is important to note that all recruitment message and study measures were only presented in the English language, thus, non-English speakers likely did not respond. It is unknown as to the level of English proficiency the participants had; however, analyses of internal consistency estimates are consistent with samples from previous investigations (e.g., Ryan et al., 1997) suggesting acceptable reliability of responses. This pool does not do justice to the many racial, sexual, geographical, and cultural differences within the BJJ community—especially among non-English speakers. Future researchers may wish to consider using multi-lingual translations of study instruments—most notably Portuguese (which is the native language in Brazil).

A second limitation to this study is the lack of qualitative data. While quantitative data provides a large dataset of numerical data, studies of this nature do a poor job of understanding the “why” behind participants’ responses. With a topic such as motivation, it is important to hear from participants, in their own words, their purpose and motivation to participate in their sport. An inclusion of some qualitative measure should be added to dive deeper into the personal beliefs and reasonings behind athletes’ motivations to participate in their sport.

While the concerns of blue belts within BJJ was previously addressed, participants were not to identify their belt rank within this study. Data were derived from a larger study of combat sport athletes—many of which were from sports without a belt ranking system. Future studies would greatly benefit from gathering this information to further study the influence of motivation within and across belt ranks within BJJ.

## Conclusion

Motivation is a key foundation to the actions and behaviors that an athlete partakes in with their sport. This drive and direction of motivation can be influenced by a multitude of elements that an athlete faces in their daily lives. The athlete’s motivation lies upon a spectrum between extrinsic and intrinsic motivation, depending upon the motivators driving them. BJJ players have been found to have a multitude of motivators that drive their motivation, however, there have been few studies that have provided a motivational profile for these athletes. The results of this study found that while BJJ players value both extrinsic and intrinsic motivators, there is a relative difference among motivators with competence being a driving motivator for those with goals of competing in their sport. From a practical perspective, these findings may aid practitioners, instructors, and clinicians in their approach to training and interventions with BJJ athletes.

## Data availability statement

The raw data supporting the conclusions of this article will be made available by the authors, without undue reservation.

## Ethics statement

The studies involving humans were approved by University of Tennessee, Knoxville Institutional Review Board. The studies were conducted in accordance with the local legislation and institutional requirements. The participants provided their written informed consent to participate in this study.

## Author contributions

TT and JL aided in the design of the study, data collection, and data analysis. TT was primarily responsible for writing the introduction and discussion. JL was primarily responsible for writing the method and results sections. All authors contributed to the article and approved the submitted version.

## Conflict of interest

The authors declare that the research was conducted in the absence of any commercial or financial relationships that could be construed as a potential conflict of interest.

## Publisher’s note

All claims expressed in this article are solely those of the authors and do not necessarily represent those of their affiliated organizations, or those of the publisher, the editors and the reviewers. Any product that may be evaluated in this article, or claim that may be made by its manufacturer, is not guaranteed or endorsed by the publisher.
